# Statistical significance and clinical significance

**DOI:** 10.1590/1516-3180.2014.1322817

**Published:** 2014-04-01

**Authors:** Alessandro Wasum Mariani, Paulo Manuel Pêgo-Fernandes

**Affiliations:** I MD. Thoracic Surgeon, Instituto do Coração (InCor), Hospital das Clínicas (HC), Faculdade de Medicina da Universidade de São Paulo (FMUSP), São Paulo, Brazil; II BrazilMD, PhD. Associate Professor, Discipline of Thoracic Surgery, Instituto do Coração (InCor), Hospital das Clínicas (HC), Faculdade de Medicina da Universidade de São Paulo (FMUSP), São Paulo, Brazil

Medicine is evolving faster than ever and the web and communication channels, among other technological improvements, facilitate the capacity for knowledge generation and knowledge diffusion, not only in the field of medicine but also for science in general. The form of knowledge diffusion that is used most is the publication of scientific articles. In this modern scenario, the ability to read and interpret medical articles is more than desirable: it is fundamental for up-to-date medical practice.

One important issue is the critical judgment of any study conclusion. We are already used to looking at the methods and results in order to evaluate whether they support the conclusion. However, even a well-designed and properly conducted study with a statistical significant P-value does not always imply real clinical significance. At first glance, we may ask ourselves: how could this be possible? How could our perfect positive study not be useful in clinical practice?

There are some tricks and pitfalls that could explain this:

First of all, it needs to be clear what the concepts behind these terms are. The crude statistical significance associated with the P-value means that from the statistical point of view, the study result was not due to chance. In other words, if we replicate the study, there is a probability lower than the defined critical value (e.g. for a P-value < 0.05, the probability will be less than 5%) that the result will not be the same. On the other hand, the concept of clinical significance, also called clinical importance, can be summarized as a difference between two therapy results that is large enough to justify changing the standard of care.^1^

The pitfall could be that, even if the study outcome is statistically supported, the difference may be too small to lead to a decision to change the current clinical practice. One hypothetical example would be a study to test a new drug for arterial hypertension that has a statistically significant P-value, e.g. P = 0.001. However, the blood pressure reduction is only 5 mmHg, which in clinical practice does not justify adopting the new drug. The problem in this example was not in the statistical design but in the setup of the outcome.

If the opposite occurs, i.e. a real clinical difference between groups that a significance test fails to identify, we have a type II error (failure to reject a false null hypothesis). However, type II errors can be predicted by conducting a power analysis prior to conducting the investigation. An adequate sample size reduces occurrences of type II errors. On the other hand, occurrences of type I error (incorrect rejection of a true null hypothesis) can be diminished by lowering the alpha (meaning the level of significance at which the P-value will be compared).

One thing that could be useful for establishing adequate clinical significance is to evaluate the confidence interval (CI), which includes all values between the limits. CIs are most frequently reported at the 95% confidence level, which means that there is a 95% chance that the real mean difference is encapsulated within the upper and lower limits. If the CI includes zero, this could be interpreted as evidence that the real difference between population means is zero and that the treatment reported is not having any effect.^2^ However, the Guidelines for Reporting Statistics from the American Physiological Society state the following: "if either bound of the confidence interval is important from a scientific perspective, then the experimental effect may be large enough to be relevant".^3^

Another method, which may be helpful, is the alternative approach of the number needed to treat (NNT), which was introduced by Laupacis et al. in 1988. This method consists of summarizing the effect of treatment in terms of the number of patients that need to be treated with the therapy in order to expect to prevent one adverse event.^4^ As pointed out by Cook and Sackett, the NNT is becoming widely used as a tool for therapeutic decision-making because it is easier to interpret than the arguably less intuitive probabilities.^5^

Ultimately, in order to choose among different treatments, clinical physicians have to consider not only the P-value of the latest published paper, but also the magnitude of benefit of each treatment, side-effect profiles, direct and possibly indirect costs, patients' preferences and even their own comfort with prescribing a new therapy.

This brings us to the conclusion that the ability to understand the statistics behind articles is not enough. Having a good notion of what real clinical significance is or could be is crucial for correct interpretation of the modern medical literature. This ability to interpret clinical significance must come from the experience of clinical practice in association with an understanding of some research concepts like study power, type I and type II errors, bias, confidence interval, treatment effect and number needed to treat.
